# Supplementary material for: Causal Relationship between Aging and Anorexia Nervosa: A White-Matter-Microstructure-Mediated Mendelian Randomization Analysis
Source: Biomedicines. 2024 Aug 16;12(8):1874. doi: 10.3390/biomedicines12081874 (PMC11351342; doi:10.3390/biomedicines12081874)
Supplement: Supplementary file 1 [file biomedicines-12-01874-s001.zip › Supplementary Figure/Figure S4/Singe SNP plot of MR analysis for TL to ubm-b-1632.pdf]

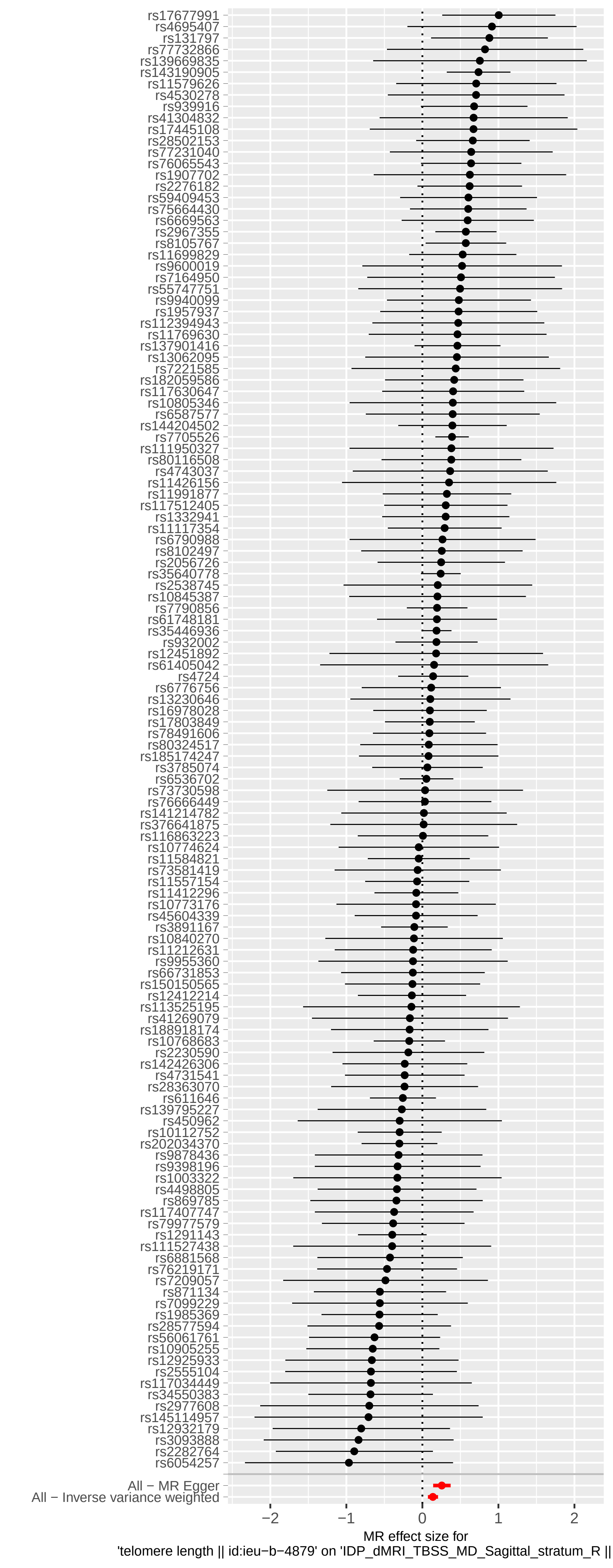
rs17677991
rs4695407
rs131797
rs77732866
rs139669835
rs143190905
rs11579626
rs4530278
rs939916
rs41304832
rs17445108
rs28502153
rs77231040
rs76065543
rs1907702
rs2276182
rs59409453
rs75664430
rs6669563
rs2967355
rs8105767
rs11699829
rs9600019
rs7164950
rs55747751
rs9940099
rs1957937
rs112394943
rs11769630
rs137901416
rs13062095
rs7221585
rs182059586
rs117630647
rs10805346
rs6587577
rs144204502
rs7705526
rs111950327
rs80116508
rs4743037
rs11426156
rs11991877
rs117512405
rs1332941
rs11117354
rs6790988
rs8102497
rs2056726
rs35640778
rs2538745
rs10845387
rs7790856
rs61748181
rs35446936
rs932002
rs12451892
rs61405042
rs4724
rs6776756
rs13230646
rs16978028
rs17803849
rs78491606
rs80324517
rs185174247
rs3785074
rs6536702
rs73730598
rs76666449
rs141214782
rs376641875
rs116863223
rs10774624
rs11584821
rs73581419
rs11557154
rs11412296
rs10773176
rs45604339
rs3891167
rs10840270
rs11212631
rs9955360
rs66731853
rs150150565
rs12412214
rs113525195
rs41269079
rs188918174
rs10768683
rs2230590
rs142426306
rs4731541
rs28363070
rs611646
rs139795227
rs450962
rs10112752
rs202034370
rs9878436
rs9398196
rs1003322
rs4498805
rs869785
rs117407747
rs79977579
rs1291143
rs111527438
rs6881568
rs76219171
rs7209057
rs871134
rs7099229
rs1985369
rs28577594
rs56061707
rs10905255
rs12925933
rs2555104
rs117034449
rs34550383
rs2977608
rs145114957
rs12932179
rs3093888
rs2282764
rs6054257
All − MR Egger
All − Inverse variance weighted
−2
−1
0
1
2
MR effect size for
'telomere length || id:ieu−b−4879' on 'IDP_dMRI_TBSS_MD_Sagittal_stratum_R ||
